# Determining the Suitability of MinION’s Direct RNA and DNA Amplicon Sequencing for Viral Subtype Identification

**DOI:** 10.3390/v12080801

**Published:** 2020-07-25

**Authors:** Deborah M. Leigh, Christopher Schefer, Carolina Cornejo

**Affiliations:** WSL Swiss Federal Research Institute, Zürcherstrasse 111, 8903 Birmensdorf, Switzerland; christopher.schefer@wsl.ch (C.S.); carolina.cornejo@wsl.ch (C.C.)

**Keywords:** MinION, RNA, cDNA, DNA amplicon, diagnostics, mycovirus

## Abstract

The MinION sequencer is increasingly being used for the detection and outbreak surveillance of pathogens due to its rapid throughput. For RNA viruses, MinION’s new direct RNA sequencing is the next significant development. Direct RNA sequencing studies are currently limited and comparisons of its diagnostic performance relative to different DNA sequencing approaches are lacking as a result. We sought to address this gap and sequenced six subtypes from the mycovirus CHV-1 using MinION’s direct RNA sequencing and DNA sequencing based on a targeted viral amplicon. Reads from both techniques could correctly identify viral presence and species using BLAST, though direct RNA reads were more frequently misassigned to closely related CHV species. De novo consensus sequences were error prone but suitable for viral species identification. However, subtype identification was less accurate from both reads and consensus sequences. This is due to the high sequencing error rate and the limited sequence divergence between some CHV-1 subtypes. Importantly, neither RNA nor amplicon sequencing reads could be used to obtain reliable intra-host variants. Overall, both sequencing techniques were suitable for virus detection, though limitations are present due to the error rate of MinION reads.

## 1. Introduction

The MinION (Oxford Nanopore Technologies Ltd., Oxford, UK, hereafter ONT) has shown it has the potential to revolutionize diagnostic protocols and pathogen surveillance. This is thanks to the device’s portability, low cost, and short sequencing time relative to other high-throughput sequencers [1]. Initially utilized in high-profile human disease outbreaks (e.g., Ebola, [2,3]; Salmonella, [4]), the MinION was shown to support rapid in situ pathogen detection and disease surveillance. More recently, the MinION has been used for smaller scale outbreaks and to detect non-human pathogens [5,6]. In a clear example of the device’s full potential for routine diagnostics, harmful DNA viruses of Cassava were confirmed within a crop in less than three hours by MinION sequencing. Amazingly, all steps leading to this diagnosis could be successfully conducted in the field [6]. Nevertheless, the error rate present in MinION reads remains significantly higher than other high-throughput sequencers (95% modal accuracy for MinION R9 reported by ONT in 2020) and this likely prevents its use in routine diagnostics.

For the detection of biologically and commercially important RNA viruses, ONT’s newly available direct RNA sequencing protocol could be a significant diagnostic advance by circumventing the need for error prone and time-consuming cDNA synthesis and PCR amplification [7,8,9,10]. In this Special Issue, direct RNA sequencing has been shown to be suitable for generating a near full-length consensus sequence of the agricultural pathogen Porcine Reproductive and Respiratory Syndrome Virus (PRRSV) and was shown to produce sufficiently accurate data to distinguish viral strains with 20 to 40% sequence divergence [11]. However, direct RNA sequencing is still in its infancy, and further explorations of the error rate and capabilities of this new technology are necessary (though, see the recent work [12]). Particularly, comparisons relative to more established DNA-based sequencing methods, such as amplicon sequencing, are needed. As well as, comparisons between more closely related viruses where the high error rate from MinION may overwhelm biological differences.

Chestnut blight (*Cryphonectria parasitica*) is an invasive cosmopolitan fungus from Asia [13]. Introduced into North America and Europe in the early 20th century, Chestnut blight has had devastating effects on the American Chestnut (*Castanea dentata*) [14] and more moderate effects within Europe (*Castanea sativa*) [13,15]. The reduced impact of the fungus in Europe is due to natural biocontrol from a fortuitously co-introduced RNA virus: *Cryphonectria hypovirus 1* (CHV-1) [15]. CHV-1 is a natural hyperparasite of the Chestnut blight fungus, belonging to a small clade of *C. parasitica* mycoviruses that includes the closely related CHV-2, CHV-3, and CHV-4 [16]. These viruses fall within the expanding viral family, *Hypoviridae*, e.g., [17]. Six different CHV-1 subtypes have been identified across Europe. All are represented in culture collections at the Swiss Federal Research Institute WSL (Birmensdorf, Switzerland). CHV-1 subtype I is the most widespread and is found along the Eastern Mediterranean [18]. The remaining subtypes are present in more localized populations: subtype F1 and F2 are found in France, E within Spain, D in Germany, and G within Georgia [18,19,20]. While the six subtypes have varying impacts on their fungal hosts and differing biocontrol potential, e.g., [21], all subtypes mitigate infection severity and reduce host tree mortality [13]. CHV-1 monitoring currently uses fungal culture phenotyping and Sanger sequencing of short DNA amplicons, e.g., [19]. While this approach is reliable, MinION’s direct RNA sequencing or sequencing of viral DNA could provide diagnostic information more quickly and in greater detail than currently available.

In this study, we aimed to understand the advantages and disadvantages of ONT’s direct RNA and DNA amplicon sequencing for diagnostics using CHV-1 as a model system. We began our evaluation by confirming if we could identify CHV-1 presence from sequencing reads. While ONT direct RNA sequencing data have recently been shown to be sufficient to identify viral strains 20–40% divergent [11], CHV-1 subtypes within Europe range from 12% to only 2% divergent across the entire genome (see Results). Therefore, we also examined if it was possible to distinguish between the six closely related CHV-1 European subtypes at the sequencing read and consensus sequence level. Finally, we examined the reliability and repeatability of variant calls within each library because intra-host information is often considered an advantage of using high-throughput sequencing methods over traditional approaches [22]. Throughout our analysis, we chose the most rapid and simple analytical tools to mirror what is likely to occur in diagnostic laboratories with time sensitive analysis and limited bioinformatics expertise.

## 2. Materials and Methods

### 2.1. Isolation of Double Stranded CHV-1 RNA

*Cryphonectria parasitica* isolates infected with one of six CHV-1 focal subtypes (Table 1) were grown for five days at 25 °C in 100 mL of liquid medium (16 g D-Glc, 4 g yeast extract, FeCl3 1% 8 drops, Knop’s solution (10×) 80 mL, 800 mL H_2_O). Fungal mycelium was then harvested with a suction filter, lyophilized overnight, and frozen at −20 °C for storage. Before extraction, the dried frozen mycelium was ground in a swing mill (Retsch, MM400, Haan, Germany) using a 2 mm acid-cleaned metal bead. The replicative double stranded form of CHV-1 RNA was then extracted from 8–10 mg of the ground mycelium with the Double-RNA Viral dsRNA Extraction Mini Kit (iNtRON Biotechnology, Seongnam-Si, South Korea). Extractions followed the manufacturer’s protocol. To facilitate the lysis of the fungal cells, the mycelium powder was dissolved in a 1.5 mL tube and larger fragments were broken up by a micro pestle after the addition of the iNtRON pre-buffer. Final concentrations were measured with a Qubit RNA Assay Kit (v3.0 Thermo Fisher Scientific, Loughborough, UK). Presence of CHV-1 was then confirmed by gel electrophoresis and dsRNA stored at −20 °C.

### 2.2. Direct RNA Sequencing Library Construction

A total of ~500 ng of RNA in a volume of 9 µL was used for each sample for the construction of the RNA sequencing library. To meet this requirement, a few samples were concentrated using an isopropanol precipitation with sodium chloride (described in Thermo Fisher’s RLM RACE protocol). The library was prepared following the direct RNA sequencing protocol from ONT for MinION (SQK-RNA002 ONT, Oxford, UK). Since CHV-1 is an RNA virus with a double strand replicative form, before beginning the protocol, all samples were denatured by heating for two minutes at 100 °C, then, snap cooled on ice for two minutes. A minor modification was made to the ONT protocol to help with RNA recovery during the bead purification steps: tubes were mixed gently by flicking only and freshly made 80% EtOH was used for bead washing. A positive control was added during library preparation (Yeast Enolase II 1.3 kilobase (kb) transcript).

### 2.3. DNA Amplicon Sequencing Library Construction

The DNA amplicon sequencing library was prepared following ONT’s cDNA sequencing kit protocol (SQK-PCS108 ONT, Oxford, UK). Before cDNA synthesis, extracted RNA was denatured as above, for two minutes at 100 °C, then, snap cooled on ice for two minutes. After cooling, we immediately began first strand cDNA synthesis using the Maxima H Minus Reverse Transcriptase (Thermo Fisher Scientific, Loughborough, UK) and Oligo(dT)_12–18_ primers (Thermo Fisher Scientific, Loughborough, UK). The standard MinION protocol for second strand cDNA synthesis was unsuccessful despite several attempts, and the single strand cDNA was used directly for PCR. The full CHV-1 genome length could not be amplified due to PCR limitations and primer design constraints. Instead, we amplified three and five kilobase (kb) amplicons targeting ORFA using the high fidelity PrimeSTAR GXL DNA Polymerase (Takara, Japan). The forward primer sequences used were identical (5′-ATC YGG AGA ARG TGA TTT GC-3′), but the reverse primers targeted different genome regions (3 kb amplicon 5′-AGA YGA YGC TGG TAA ATG AAG-3′; 5 kb amplicon 5′-YTT RTT GAT GTA GCT GCG AGG-3′). The two amplicons were used to provide a technical replicate library for each sample. In total, 30× PCR cycles were used for each primer pair. The two PCR reactions of each CHV-1 strain were then pooled and cleaned with Agencourt RNAClean XP beads (Beckman Coulter, Brea, CA, USA). MinION’s end-prep, barcoding, and adapter ligation were performed on the pooled products with the barcode expansion EXP-NBD103. Further modifications to ONT’s protocol were made during bead purification. Binding of DNA to the RNAClean XP beads was elongated to 10 min. Beads were also incubated at 37 °C for 15 min during the elution of the purified DNA to increase yield. The final amount of (pooled) dsDNA in the library was between 300–650 ng. It should be noted that the PCR primers failed to amplify one of our more divergent CHV-1 subtypes, G. This prevented us from sequencing this subtype with a DNA amplicon library.

### 2.4. Sequencing Conditions for the MinION

Sequencing was performed in-house at WSL (Phytopathology, Birmensdorf, Switzerland). For RNA sequencing, each library was loaded onto a MinION R9.4 flow cell on a MinION Mk1B device (ONT) and sequenced for 8–12 h. Failed runs were identified and excluded at this point. DNA libraries were also sequenced using a MinION R9.4 flow cell on a MinION Mk1B device (ONT) for 18 h. The MinKNOW software v.2.0 (ONT) was adjusted according to ONT’s sequencing protocol with live basecalling disabled. The DNA amplicon library flow cell was used at least two times, with the 5 kb library run first, followed by the 3 kb library (voltage was adjusted according to ONT’s washing protocol). Basecalling was performed with Guppy (v2.3.5 ONT).

### 2.5. Direct RNA Sequencing Read Processing

Direct RNA sequencing reads were filtered to remove reads belonging to the kit positive control with NanoLyse (v1.1.0 [23], reference accession number NC_001140.6). Reads were then quality filtered with NanoFilt (v2.2.0 [23]). Only reads above 2 kb and quality score (*q*) of *q* ≥ 8 were retained for downstream analysis.

### 2.6. DNA Sequencing Read Processing

Amplicon reads were demultiplexed using qcat (v1.1.0 ONT), the entire read was searched for barcodes, and all barcodes trimmed out. Reads below a minimum *q* score of 10 were then filtered with NanoFilt for both libraries. For the 3 kb amplicon DNA library, reads shorter than 2000 basepairs (bp) and longer than 4 kb were also excluded. For the 5 kb amplicon DNA library, reads shorter than 4 kb and longer than 6 kb were excluded.

### 2.7. CHV-1 Subtype Identification from Filtered Reads

Three different CHV-1 subtypes were successfully sequenced using direct RNA sequencing (runs for two subtypes failed to produce data and were excluded from our analysis) and five different subtypes with DNA amplicon sequencing (Table 1). Though the subtype was known a priori, it is important to evaluate the diagnostic potential of this technology and confirm if CHV-1 presence and subtype could be accurately inferred from sequencing reads alone. To test this, filtered RNA and DNA amplicon reads were submitted to a local BLAST search against a custom database containing CHV-1 subtype genome sequences (I, F1, F2, E, D, G), and full-length sequences of the closely related viruses CHV-2/3/4. The option max_target_seqs was set to simplify the BLAST output; this returns the first *N* ‘good’ hits in the BLAST catalogue and is sensitive to the order of sequences [24]. The order of reference sequences in the database was: I, F1, F2, D, E, G, CHV-2, CHV-3, and CHV-4. The top BLAST hit for each sequencing read was then extracted and the proportion of correctly identified reads estimated. Because we expected a high error rate per read, we did not exclude hits with low BLAST alignment quality scores, though the mean read percentage identity for each hit was recorded. To gain understanding of the sequence level divergence of subtypes and help interpret the BLAST analysis, we also compared the available full genome reference sequences, as well as the corresponding 3 and 5 kb amplicon regions in CLC workbench 7 (v10.1.1 Hilden, Germany, QIAGEN) using the pairwise comparison tool.

### 2.8. CHV-1 Consensus Generation

Consensus sequences have been used for pathogen identification in diagnostic studies and we explored if they offered greater accuracy than read-based methods. To generate a consensus sequence from each library type, we began by assembling de novo with Wtdbg2 (v2.5 [25]). A de novo assembly approach was taken because it is assumption free about the pathogen present and will not generate any bias towards the initial reference sequence used. Furthermore, it enables us to examine the accuracy of species and subtype identification when the virus or subtype present is unclear. For both the DNA amplicon and the direct RNA sequencing reads, Wtdbg2 was run on all uncorrected reads to reduce processing time, assuming a genome size of 12.7 kb and the ‘ont’ setting suitable for error prone ONT reads. We included all reads, including those that did not have CHV-1 as a top BLAST hit. The DNA libraries were PCR amplicons and, therefore, additional Wtdbg2 parameter adjustment was necessary. Accordingly, our repetitive sequence (‘K’) filter was increased to 100 thousand reads. Furthermore, to ensure an output consensus was generated for low quality samples, the minimum number of nodes allowed in a contig was reduced to two and the minimum length of a contig reduced to 1 kb. For the 3 kb libraries, it was also necessary to reduce to read length filter to 1 kb and to increase the maximum node depth to 500 reads. These parameter changes may reduce consensus quality [25], but were necessary for consensus to be produced and may still improve our pathogen identification accuracy. The longest consensus fragment was taken for downstream processing. These were used to identify subtype following the BLAST search approach detailed above. In addition, consensus sequences from the RNA libraries were imported into MegaX (v10.1.2 [26]) and aligned with Muscle (default settings with 50 replicates) [26,27]. A phylogeny was then constructed using a Maximum Likelihood tree and 500 bootstraps replicates with consensus sequences and pre-existing reference sequences. This allowed us to evaluate if subtypes sequenced with direct RNA sequencing could be correctly identified through a very simple and rapidly produced phylogeny. Only RNA consensuses sequences were used for this analysis as they are genome-wide, while the amplicon libraries were not.

### 2.9. Repeatability of Variant Calls

To call variants, sequencing reads were mapped to a CHV-1 reference genome listed in Table 1. Reads were mapped using Minimap2 (v2.17 [28]) with the recommended settings for MinION reads. For the RNA libraries, Canu corrected reads for this analysis because of the lower *q* score filter needed to obtain sufficient data. The SNP callers used included: AssociVar (v1 [29]), iVar (v1.0.1 [30]), Ococo (v0.1.2.7 [31]), and FreeBayes (v1.3.1 [32]). iVar was run without base quality alignment and the following filters: a minimum base and mapping quality of 20, a minimum variant quality score of 30, and a frequency of 0.2. Ococo and AssociVar were run using the default settings. FreeBayes was run assuming a ploidy of 1, with the flags pooled discrete and pooled continuous set. To reduce memory requirements, variants were only evaluated by FreeBayes if they had a minimum base quality of 30, a maximum of 2 alleles per site, and were seen on 20 or more reads.

## 3. Results

### 3.1. Run Statistics

For the direct RNA sequencing libraries: 15,358 reads were produced for subtype G, with a mean *q* score of 9.1 and mean read length of 4.6 kb. Subtype I had 6283 reads produced, with a mean *q* score of 9.1 and a mean read length of 3.4 kb. For subtype F1, 7251 reads were produced with a mean *q* score of 9 and a mean read length of 2.9 kb. For the DNA amplicon libraries, over 2 million reads were produced for the 5 kb library with a mean read *q* score of 9.5 and mean read length of 4.5 kb. For the 3 kb library, 2.8 million reads were produced, with an average length of 2.7 kb and an average read quality of 9.6.

### 3.2. CHV-1 Subtype Identification

Reads from the direct RNA and DNA amplicon sequencing libraries were submitted to a BLAST search against the CHV custom reference database. The largest proportion of reads had a top hit belonging to any CHV-1 subtype (Table 2), indicating that identification of viral species was possible. However, identification of the correct viral subtype had more varied success. Subtypes with lower pairwise sequence divergence, E and D, could not be distinguished in the 3 kb amplicon library. The pairwise percentage identity and sequence divergence for subtypes are shown in Table 3. E and D are the most closely related subtypes.

### 3.3. Consensus Sequence Accuracy

**Figure 1:**
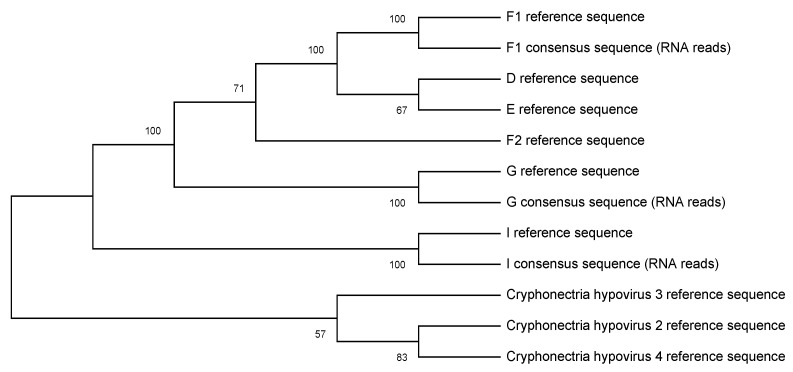
Shown is a maximum likelihood phylogeny estimated in MegaX. Included are the reference sequences for each subtype and CHV species, as well as the consensus sequences from Wtbdg2 for each subtype sequenced with direct RNA sequencing.

Consensus sequences for each sequencing library were also subject to a BLAST search in the same manner detailed above; this yielded identical results to the sequencing reads (Table 2). The length of the longest consensus sequence for each library ranged from 2995 to 4265 bp for the 3 kb amplicons (23–33% genome-wide coverage), and 4905–6757 bp for the 5 kb amplicons (38–53% genome-wide coverage). All consensus sequences were close to their expected sizes of 3 and 5 kb; some exceeded this due to reads present from outside the target region. For the direct RNA sequencing libraries, the longest consensus sequences were 7921 bp (F1, 63% genome-wide coverage), 12,018 bp (I, 94% genome-wide coverage), and 12,144 bp (G, 97% genome-wide coverage). The phylogeny drawn in MegaX using the full-length consensus sequences from our direct RNA reads only, matched biological expectations (see [20]). This could be used to identify subtypes through looking at sister species (Figure 1).

### 3.4. Repeatability of Variant Calls

To detect within subtype mutations, four variant callers were applied to the filtered and aligned ONT reads. FreeBayes repeatedly failed due to high memory requirements (>100 GB). Furthermore, the MinION-specific variant caller AssociVar failed to produce an output after two weeks. Both programs were excluded from further analysis. The number of variants called by Ococo and iVar is shown in Table 4. Each sequencing library had a high number of private variants. A low proportion of the variants identified using Ococo and iVar were consistent across the RNA and DNA libraries (Table 4). Furthermore, an average overlap of only 5% (±9% standard deviation) was found when overlapping variants from the same library called across the two softwares.

## 4. Discussion

In this study, we explored the suitability of ONT’s direct RNA sequencing and DNA amplicon sequencing for detecting CHV-1 presence within Chestnut blight fungal cultures. Viral presence and species could be correctly identified using BLAST searches of raw sequences and consensus sequences. Subtype was more difficult to correctly infer and required longer (>3 kb) consensus fragments to be identified correctly. Intra-host variant calls were not repeatable across libraries. Importantly, two direct RNA sequencing runs failed entirely due to the difficulties in applying this method.

### 4.1. Identifying Species with Different ONT Read Types

The fundamental objective of any diagnostic study is confirming the presence of a pathogen within a sample. In this study, a BLAST search of filtered reads from direct RNA and DNA amplicon sequencing libraries correctly identified CHV-1 within a sample. Despite the high technical error rate expected from ONT data, CHV-1 could be distinguished from closely related mycoviruses that can also occur in Chestnut blight cankers. However, direct RNA sequencing reads were misassigned more frequently than DNA sequencing reads. Only a small difference in percentage error rate was expected between RNA and DNA reads based on the *q* score filters (*q* score 8 vs. 10, <5% difference based on [33]). Consequently, differences in library structure, i.e., amplicon vs. whole genome, may be driving the increased misassignment probability. Nevertheless, due to the non-negligible misassignment rate of direct RNA reads, direct RNA reads should not be used by methods requiring species identification from individual reads, such as characterizing a virome (e.g., [34]), because of the risk of species misidentification. Though good species assignment accuracy was possible for DNA amplicons reads, the intrinsically high MinION error rate also makes individual read-based virus identification unsuitable for diagnostics. Threshold read numbers or proportions, similar to Ct cutoffs [35], should be used to confirm viral presence with DNA reads.

Consensus sequences were extremely reliable for species identification and were always correctly identified as CHV-1 through BLAST, even though the DNA sequences were based on amplicon libraries. These results add to the growing body of evidence that viral species can be accurately identified using consensus sequences from DNA and direct RNA sequencing reads (influenza, [36]; PRRSV, [11]).

### 4.2. Identifying Closely Related Subtypes with Different ONT Read Types

Distinguishing between closely related CHV-1 subtypes had variable success and was closely linked to the biological distances of subtypes. Subtype could be correctly identified across reads and consensus sequences for all but two DNA amplicon libraries. The 3 kb libraries from subtype E and D were misassigned to each other; however, the correct focal subtype could be identified using the 5 kb libraries. Subtype D is a putative recent recombinant of subtypes E and I [37]. Previous studies have also struggled to split subtype E and D based on a small fragment of ORFA, and required additional sequences from ORFB to do so [37]. Due to primer constraints, neither amplicon includes the region of ORFB used previously. However, the 3 kb amplicon does include 400 bp from ORFB and the 5 kb amplicon covers nearly 2.7 kb of ORFB. For the 3 kb amplicon, this was likely an insufficien portion of ORFB or an insufficiently divergent section of ORFB to distinguish E and D. Due to D’s recombinant origin, subtypes are very closely related at the sequence level, with close to 2% sequence divergence genome-wide. Comparisons of the amplicon regions show the same estimate of 2% across the 5 kb amplicon, but less than 1% for the 3 kb amplicon. Consequently, there is very limited biological variation in the 3 kb amplicon to distinguish these two subtypes. This variation is likely insufficient when coupled with MinION’s error rate to distinguish between subtypes. This result highlights that longer amplicon targets, or more divergent targets (>2%), are necessary for studies seeking to distinguish between closely related subtypes with MinION DNA amplicon data.

For the direct RNA sequencing libraries, all three subtypes were correctly identified through the BLAST analyses of the reads and consensus sequences. The subtypes sequenced were between 4–12% divergent from other subtypes in the reference database. It must be noted that subtype identification accuracy was dependent on a full reference sequence being available a priori. Many reads were misassigned to closely related subtypes, thus, care must be taken when working with RNA sequencing data from new strains or subtypes to ensure that they are not misidentified as close relatives in the catalogue. For this reason, we recommend that researchers couple a BLAST search with a phylogeny. This will confirm if consensus sequences follow our biological expectations and may help identify subtypes where only genome fragments are available a priori. Furthermore, BLAST catalogues must be examined thoroughly before performing a read or consensus BLAST analysis to ensure sequences are correctly labeled. For CHV-1, the full-length sequence CHV-1 subtype G is present in NCBI but is incorrectly classed as subtype F2 [38]. This a historical misidentification that arose because subtype G is a recombinant of subtypes F2 and D [20]. This misidentification could have easily led to incorrect read assignment and highlights the importance of database curation.

Two additional RNA libraries were sequenced within this study and failed to produce sufficient data for analysis. The challenges associated with applying a new technology should not be ignored by future studies seeking to use direct RNA sequencing. Failed sequencing runs and delays must be incorporated into study designs. This may limit direct RNA sequencing’s suitability for studies requiring a rapid result, when in-house protocols have not been established.

### 4.3. Repeatability of Variant Calls from MinION Data

In this study, we reconfirmed that MinION data are currently too error prone for accurate variant calling. We found a low repeatability of variant calls across both sequencing techniques and across the 3 and 5 kb amplicon libraries. This result is in line with many previous studies (e.g., [30]), and MinION data should not be used for variant calling until sequencing error rate is reduced [39].

## 5. Conclusions

In this study, we showed that MinION’s direct RNA and DNA sequencing reads and consensus sequences can both be used to identify viral species and distinguish between subtypes. However, direct RNA sequencing reads show a high species misassignment rate when examined independently and should not be used to characterize complex samples with several viral species present. Furthermore, a long read length and sufficient biological differences relative to the expected error rate were needed to distinguished closely related subtypes. Consequently, MinION reads or consensus alone will likely be insufficient to definitively confirm the presence of viruses with many closely relatives or limited biological information a priori. Furthermore, reliable intra-host variants could not be called across either sequencing technique and MinION data should not be used for this purpose until the error rate is reduced. While the diagnostic potential is promising, many challenges remain when using MinION sequences and cannot be ignored in diagnostics, where accuracy is essential.

## Figures and Tables

**Table 1:** The CHV-1 subtypes sequenced as DNA amplicons or directly as RNA. Subtypes each have two IDs: their viral subtype and the WSL laboratory strain ID. Also shown is the accession number in GenBank for the reference sequences used for each subtype.

CHV-1 Subtype	WSL Collection Number	MinION Libraries Sequenced	Reference Sequence GenBank Accession Number
G	CHV1-M7757	RNA	MF421719.1 ^+^
I	CHV1-EP721	RNA, 3 kb DNA, 5 kb DNA	DQ861913
F1	CHV1-M1123	RNA, 3 kb DNA, 5 kb DNA	NC_001492
F2	CHV1-M2021	3 kb DNA, 5 kb DNA **	MF421718
D	CHV1-M1372	3 kb DNA, 5 kb DNA	MF431594
E	CHV1-M9291	3 kb DNA, 5 kb DNA **	MF431593

** RNA sequencing attempted, run failed. ^+^ misidentified as F2 in GenBank.

**Table 2:** BLAST analysis for raw sequencing reads. Shown are the number reads with the top BLAST hit to CHV-1 or other CHV species for each sequencing library. PI—mean percentage identity of the top hits. Underlined values denote an incorrect subtype assignment.

CHV-1 Subtype.	Sequencing Library	Reads with a Top BLAST Hit to the Correct CHV-1 Subtype (PI)	CHV-1 Subtype with the Highest Proportion of Reads	Reads with a Top BLAST Hit to Any CHV-1 Subtype (PI)	Reads with Top BLAST Hit to Other CHV Species (PI)	Consensus Sequence Top BLAST Hit (PI)
**G**	RNA	52.03% (PI: 90.82)	G	70.36% (PI: 91.79)	29.64% (PI: 95.0)	G (PI:96.01)
**I**	RNA	29.76% (PI: 90.61)	I	61.2% (PI: 92.71)	38.7% (PI: 94.93)	I (PI:90.99)
	3 kb DNA	99.68% (PI: 90.02)	I	99.99% (PI: 92.02)	8.08 × 10^−6^% (PI: 88.39)	I (PI: 92.43)
	5 kb DNA	99.67% (PI: 93.68)	I	99.99% (PI: 93.68)	4.4 × 10^−5^% (PI: 95.16)	I (PI: 96.43)
**F1**	RNA	93.78% (PI: 86.08)	F1	96.61% (PI: 86.33)	0.034% (PI: 94.92)	F1 (96.86)
	3 kb DNA	99.93% (PI: 94.86)	F1	99.99% (PI: 94.87)	1.8 × 10^−6^% (PI: 84.38)	F1 (PI: 79.48)
	5 kb DNA	99.92% (PI: 94.82)	F1	99.99% (PI: 94.81)	1.3 × 10^−6^% (PI: 94.12)	F1 (PI: 99.48)
**F2**	3 kb DNA	99.92% (PI: 92.68)	F2	99.99% (PI: 92.68)	2.5 × 10^−6^% (PI: 94.44)	F2 (PI: 98.20)
	5 kb DNA	99.82% (PI: 94.12)	F2	100% (PI: 94.12)	0	F2 (PI: 96.02)
**D**	3 kb DNA	0.0003% (PI: 93.06)	E (99.69% of reads)	100% (PI: 89.25)	0	E (PI: 86.95)
	5 kb DNA	94.17% (PI: 91.16)	D	99.99% (PI: 91.04)	2.0 × 10^−5^% (PI: 92.96)	D (PI: 91.59)
**E**	3 kb DNA	29.75% (PI: 94.36)	D (70.1% of reads)	99.99% (PI: 94.74)	1.6 × 10^−5^% (PI: 92.97)	D (PI: 90.87)
	5 kb DNA	95.7% (PI: 94.58)	E	100% (PI: 94.5)	0	E (PI: 86.61)

**Table 3:** Shown is the divergence (upper rows) and percentage identity (lower half) between full length reference sequences. In brackets are the estimates for the 3 and 5 kb amplicon regions only. Estimates were calculated using the sequences from NCBI listed in Table 1 and CLC.

		Sequence Divergence					
		**I** (3 kb/5 kb)	**F1** (3 kb/5 kb)	**F2** (3 kb/5 kb)	**D** (3 kb/5 kb)	**E** (3 kb/5 kb)	**G** (3 kb/5 kb)
**Percentage Identity**	**I**	-	0.12 (0.11/0.12)	0.10 (0.07/0.10)	0.11 (0.10/0.12)	0.11 (0.10/0.12)	0.10 (0.08/0.11)
	**F1**	89 (90/89)	-	0.08 (0.09/0.05)	0.07 (0.06/0.05)	0.06 (0.06/0.04)	0.10 (0.10/0.08)
	**F2**	89 (92/89)	91 (90/94)	-	0.05 (0.08/0.06)	0.05 (0.07/0.05)	0.04 (0.03/0.04)
	**D**	88 (89/88)	92 (92/94)	95 (93/94)	-	0.02 (0.01/0.02)	0.06 (0.09/0.06)
	**E**	88 (89/88)	93 (92/95)	95 (93/96)	98 (99/98)	-	0.06 (0.09/0.07)
	**G**	89 (91/89)	89 (89/91)	96 (97/96)	94 (91/94)	94 (91/92)	-

**Table 4:** The number of polymorphic intra-host variants for each subtype identified by the two variant callers that completed analysis, iVar and Ococo. The percentages shown are the number of overlapping variants divided by the mean number called from the focal libraries.

		Number of Variants				
	Subtype	RNA	3 kb	5 kb	RNA/DNA Overlap (Percentage Variants Overlapping)	3 kb/5 kb Overlap (Percentage Variants Overlapping)
*Ococo*	G	1145	NA	NA	NA	NA
	I	54	6	8	2 (9%)	5 (71%)
	F1	87	4	8	0 (0%)	1 (17%)
	F2	NA	13	16	NA	11 (76%)
	D	NA	210	225	NA	144 (66%)
	E	NA	17	25	NA	16 (76%)
*iVar*	G	1845	NA	NA	NA	NA
	I	670	612	1267	18 (2%)	397 (42%)
	F1	683	595	1209	17 (2%)	387 (43%)
	F2	NA	577	1101	NA	399 (48%)
	D	NA	667	1268	NA	488 (50%)
	E	NA	632	1222	NA	455 (49%)

## Data Accessibility

Consensus sequences from the RNA libraries have been uploaded to NCBI. Raw data available on request from DML and CC.
